# Technical Considerations of Ring-Augmented Laparoscopic Sleeve Gastrectomy: A Step-by-step Guide for Ring Placement by Various Experts

**DOI:** 10.1007/s11695-025-07674-2

**Published:** 2025-01-16

**Authors:** Mohamed Hany, Frits Berends, Edo Aarts, Jodok Fink, Evert-Jan G. Boerma, Bart Torensma

**Affiliations:** 1 Department of Surgery, Medical Research Institute, Alexandria University, 165 Horreya Avenue, Hadara, Alexandria, 21561 Egypt; 2 Department of Surgery, WeightWorks Clinics, Amersfoort, The Netherlands; 3 Centre for Surgery, Department of General and Visceral Surgery, Centre for Obesity and Metabolic Surgery, Medical Centre, University of Freiburg, Freiburg, Germany; 4 Department of Surgery, Zuyderland Medical Center, Heerlen, The Netherlands; 5 Clinical Epidemiologist, Leiden University Medical Center (LUMC), Leiden, The Netherlands

**Keywords:** Sleeve gastrectomy, Metabolic bariatric surgery, Ring augmentation, Ring placement restriction, Banded sleeve gastrectomy

## Introduction

Laparoscopic ring-augmented sleeve gastrectomy [1] is a technically demanding metabolic bariatric procedure that, when performed correctly, can enhance weight loss outcomes and reduce side effects of sleeve gastrectomy, such as long-term dilation of the gastric sleeve. Better weight loss outcomes are observed at 3 and 5 years postoperatively, with a margin of 6.4% and 9.9% in total weight loss (%TWL), respectively, following ring-augmented sleeve gastrectomy [2].

This video demonstrates three distinct surgical approaches performed by three different surgeons from three different countries. Each surgeon showcases their preferred technique for performing a ring-augmented sleeve gastrectomy, outlining the essential steps and providing detailed explanations for their respective procedures.

## Ring-Augmented Sleeve Gastrectomy: What Have We Learned from Each Other?

Although the surgeons reviewed multiple videos showcasing different techniques, all approaches ultimately led to the same successful outcome, demonstrating that various methods can achieve optimal ring placement. However, continuous improvement and learning from one another remain essential to refine these techniques further. Therefore, it is crucial to consider the following points to enhance procedural outcomes and standardize best practices.

The first consideration is the positioning of the ring, which should be placed approximately 3 to 5 cm below the gastroesophageal junction. This location prevents proximal sleeve dilatation above the ring and allows for relatively easy perigastric dissection and access to the lesser sac. The higher the position of the ring, the lower the risk of gastroesophageal reflux disease (GERD) because an acid barrier is created for the acid that arises from the stomach [3, 4].

Ensuring the ring is not too tight is critical for patient safety and postoperative comfort. The diameter of the sleeve and the size of the bougie used influence this from the outset. Therefore, avoiding placing the ring too loosely is more important than relying on a predefined diameter.

Furthermore, excessive tension can cause significant food intolerance and regurgitation, resulting in patient dissatisfaction and poor nutritional outcomes [5]. Leaving the ring slightly loose allows the stomach some flexibility in accommodating ingested food while still providing the necessary mechanical restriction to facilitate weight loss.

Once the ring is correctly positioned, trimming the ends is optional. Untrimmed ends allow for adjustments of the diameter of the ring at a later time if needed. However, long ends can interfere with surrounding tissues, such as the overlying liver, which could lead to irritation or adhesion formation. By cutting the ends to the appropriate length, the surgeon ensures the ring aligns smoothly with the stomach, promoting patient comfort and avoiding complications during regular digestive movements.

Securing the ring with any form of sutures is essential for maintaining its position over the long term. Although absorbable sutures are sufficient for temporary fixation, they can result in the ring coming loose as the sutures degrade, potentially leading to complications such as ring slippage. Nevertheless, non-absorbable sutures provide permanent fixation, ensuring the ring remains correctly positioned and continues functioning as intended without the risk of displacement.

Additionally, securing the staple line of the sleeve is an innovative approach, as it ensures stable fixation at the 9 o’clock position in the mesentery and the 3 o’clock position along the staple line while also preventing slippage over the sleeve lumen.

Minimizing the presence of fat between the stomach wall and the ring is also an important technical consideration. Excess adipose tissue can act as a buffer, reducing the ring’s effectiveness in creating the desired diameter and function. Moreover, as fat gradually disappears with progressive weight loss, the ring may become too wide, increasing the risk of slippage. The surgeon can maximize the ring’s effectiveness by careful perigastric positioning of the ring directly onto the gastric wall with minimal fat interference.

Lastly, whether the ring is introduced from right to left or left to right is a matter of surgical preference. This decision often depends on the surgeon’s dominant hand, the patient’s anatomy, and trocar positioning. Allowing this flexibility ensures that surgeons can perform the procedure most ergonomically and efficiently, ultimately reducing operative time and enhancing precision.

By following these expert tips and adhering to the above principles, surgeons can improve their outcomes when performing laparoscopic ring-augmented sleeve gastrectomy.

We have information from a systematic review regarding complications after laparoscopic ring-augmented sleeve gastrectomy. The meta-analysis published in 2022 by Gupta et al. included six studies—two randomized controlled trials (RCTs) and four observational studies (three prospective and one retrospective)—encompassing 660 patients. The analysis demonstrated that using a ring in sleeve gastrectomy does not notably increase significant postoperative complications. A key observation was a revision rate of 7.1%, primarily attributed to regurgitation following the application of the ring [2].

In four out of the six studies from the SR, the same type of ring was used by the three surgeons, and both the distance of the ring from the gastroesophageal (GE) junction and the ring size are similar to those demonstrated in our video. However, how the procedures were performed in the studies included in the meta-analysis remains to be seen.

Furthermore, the incidence of band erosions associated with the placement of a ring in sleeve gastrectomy (rLSG) appears to be exceedingly rare, as it has not been reported in studies or systematic reviews specifically addressing rLSG. However, a case report published by Hany et al. described a patient with laparoscopic rLSG who presented with a 3-month history of persistent vomiting, decreased tolerance for fluids, and limited intake of soft foods due to a slipped and eroded band [6].

Notably, this case involved a different type of band, similar to the older gastric bands, and it was placed in a manner distinct from the techniques currently used for ring placement in rLSG. Therefore, additional studies and case reports are needed to clarify ring erosion incidence and outcomes with modern techniques and materials.

That said, while we can assume that the three techniques demonstrated in our video—and potentially the six techniques utilized in the meta-analysis—lead to comparable outcomes, further clarity is needed. The purpose of our video is to highlight that multiple approaches are feasible for performing ring-augmented sleeve gastrectomy.

Our research group is preparing a new study in which a propensity score-matched cohort analysis will be performed. This study will systematically evaluate outcomes from the three surgeons, including weight loss and complication rates, across different techniques to determine whether variations in surgical approaches influence clinical outcomes.

## Supplementary Information

Below is the link to the electronic supplementary material.

Supplementary file1 (MP4 380316 KB)

## Data Availability

No datasets were generated or analysed during the current study.
